# Uncovering the genomic basis of lactose content in Valle del Belice dairy sheep

**DOI:** 10.3168/jdsc.2026-1046

**Published:** 2026-06-25

**Authors:** Salvatore Mastrangelo, Maria Teresa Sardina, Baldassare Portolano, Marco Tolone, Alberto Cesarani

**Affiliations:** 1 Dipartimento di Scienze Agrarie, Alimentari e Forestali, University of Palermo, Palermo 90128, Italy; 2 Dipartimento di Scienze Chimiche, Biologiche, Farmaceutiche ed Ambientali, University of Messina, Messina 98122, Italy; 3 Dipartimento di Agraria, University of Sassari, Sassari 07100, Italy; 4 Department of Animal and Dairy Science, University of Georgia, Athens, GA 30602

## Abstract

The aim of this study was to investigate the genomic background of lactose content in Valle del Belice dairy sheep. A total of 3,183 milk test-day records from 326 ewes genotyped for 39,054 SNPs were analyzed. Heritability and repeatability were estimated with an animal model considering parity as fixed cross-classified effect, days in milk as fixed covariate, and flock test-date, animal, permanent environment, and residual as random effects. The animal effect was modeled using the genomic relationship matrix among the ewes. Two approaches were applied to perform the GWAS: back-solving of genomic breeding values and a Bayesian method. Moderate heritability (0.20 ± 0.06) and repeatability (0.33 ± 0.03) were estimated, consistent with values reported in the literature. Phenotypic and genomic correlations between lactose content and the other considered milk traits were not significantly different from zero. Only one SNP, located at 33,635,275 bp on chromosome 2, exceeded the adopted genome-wide significance threshold in both GWAS approaches. This SNP mapped close to 4 annotated genes: *ISCA1*, *TUT7*, *NAA35*, and *GOLM1*. The first gene has been associated with mitochondrial function, whereas the others have been linked to immune-related processes. This study represents the first genomic investigation of lactose content in Valle del Belice sheep and suggests that this trait could be considered in the breeding objective of the breed, given its moderate heritability and repeatability.

## Graphical Abstract

**Figure:**
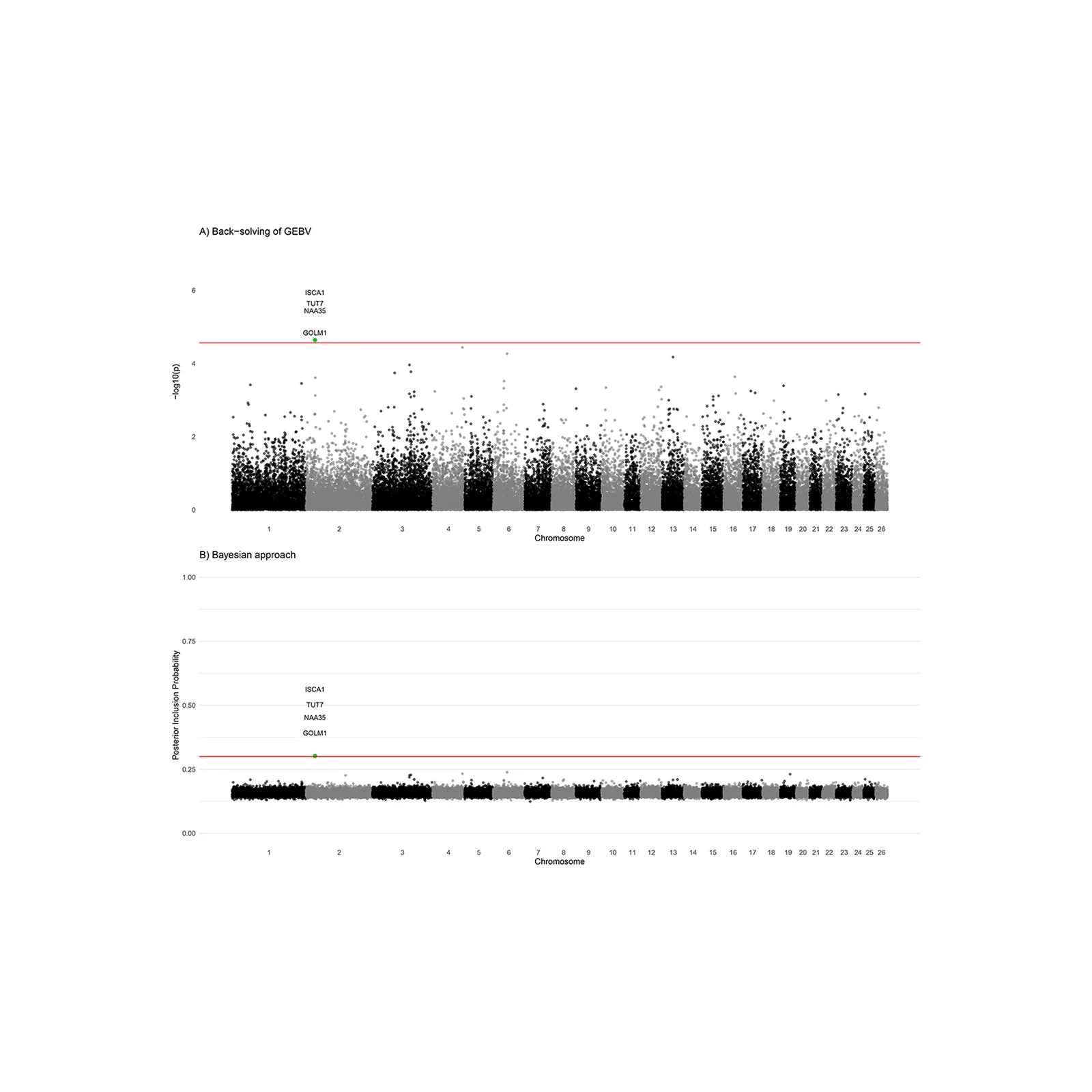

Lactose is the main carbohydrate in milk and plays a central role in regulating milk volume through osmotic balance within the mammary gland (Fox et al., 2015; Lin et al., 2016). As the primary osmotic component of milk, synthesis of lactose largely determines milk secretion by controlling water influx into the alveolar lumen (Cai et al., 2018). Beyond its physiological function, lactose content also influences milk processing properties and technological performance (Vacca et al., 2019). In addition, particular attention has been devoted to the inverse relationship observed between lactose content and SCC in dairy cattle, at both the phenotypic and genetic levels (Costa et al., 2019a), suggesting a potential association between lactose concentration and udder health. At the biological level, lactose synthesis depends on glucose availability and on the activity of lactose synthase complex in mammary epithelial cells, involving genes related to carbohydrate metabolism, glucose transport, and mammary gland regulation (Liu et al., 2013). These mechanisms indicate that breed-specific genetic architectures may underline differences in lactose content across dairy populations. From a genomic perspective, GWAS have been conducted to elucidate the genetic basis of milk lactose in cattle (Lopdell et al., 2017; Costa et al., 2019b), buffalo (Awad et al., 2020), and goat (Selionova et al., 2024). However, information specifically addressing the genomic background of lactose content in dairy sheep remains limited, particularly in Mediterranean populations (Carta et al., 2023), where local breeds play a crucial role in regional dairy systems. The Valle del Belice breed is one of the most important dairy sheep breeds in Southern Italy, characterized by high milk production and adaptation to semiextensive production systems (Mastrangelo et al., 2017; Di Gerlando et al., 2019). Despite its economic relevance and the increasing implementation of genomic tools in this breed (Sutera et al., 2019, 2021a; Ben Jemaa et al., 2023), the genetic architecture of lactose content has not yet been comprehensively investigated. A better understanding of the phenotypic variability and genomic determinants of lactose in this breed could contribute to the identification of markers associated with milk quality and health-related traits. Therefore, the aim of this study was (1) to estimate the genetic parameters for lactose content in Valle del Belice dairy sheep and (2) to identify genomic regions associated with this trait using 2 different GWAS approaches.

Phenotypic data from 421 ewes were collected in 4 flocks of Valle del Belice dairy sheep. Milk samples were collected at approximately monthly intervals, following an A4 recording scheme (ICAR, 2014) across multiple lactations of the same ewes. Milk composition (i.e., lactose, fat, protein, and casein contents) for 4,014 test-day records was determined by infrared spectrophotometry using a Combifoss 6200 system (Foss Electric, Hillerod, Denmark). All ewes were genotyped using the OvineSNP50 BeadChip (Illumina Inc., San Diego, CA). Genotype quality control was performed using PLINK v1.9 software (Chang et al., 2015) with the following criteria: genotype and animal call rates ≥95% and minor allele frequency ≥5%. Chromosomal coordinates for SNPs were updated to the ARS-UI_Ramb_v2.0 (*Ovis aries*) genome sequence assembly, and unmapped markers were removed. After quality control, 3,183 test-day records from 326 individuals (keeping only animals with at least 2 records) and 39,054 SNPs were retained for subsequent analyses.

To estimate variance components, the following repeatability animal model was used:

[1] Y = FTD + DIM + PAR + ANIM + PE + e,

where Y is the test-day records; FTD is the random cross-classified effect of the flock test-date combination (107 levels); DIM is the fixed covariate of the days in milk; PAR is the fixed cross-classified effect of the parity of the ewe (5 levels); ANIM and PE are the random effects of animal and permanent environment, respectively; and e is the residual random effect. The random effects in the model were assumed to be distributed as follows:

$$\text{\textbackslash{}[}FTD=N\left(0,I\sigma_{FTD}^{2}\right),\text{\textbackslash{}]}$$

$$\text{\textbackslash{}[}ANIM=N\left(0,G\sigma_{a}^{2}\right),\text{\textbackslash{}]}$$

$$PE=N\left(0,I\sigma_{PE}^{2}\right),$$

$$e=N\left(0,I\sigma_{e}^{2}\right),$$

where **I** is an identity matrix, **G** is the genomic relationship matrix built using method 1 proposed by VanRaden (2008); $\text{\textbackslash{}[}\sigma_{FTD}^{2},\text{\textbackslash{}]}$ $\sigma_{a}^{2},$ $\sigma_{PE}^{2},$ and $\sigma_{e}^{2}$ are the variances associated with the FTD effect, the additive genetic variance, the permanent environmental variance, and the residual variance, respectively. The contribution of the FTD effect on the total phenotypic variance (FTD^2^), the heritability (h^2^), and the repeatability (**r^2^**) were computed as

FTD2=σFTD2/σFTD2(σFTD2+σa2+σPE2+σe2)(σFTD2+σa2+σPE2+σe2),

h2=σa2/σa2(σFTD2+σa2+σPE2+σe2)(σFTD2+σa2+σPE2+σe2),

r2=(σa2+σPE2)/(σa2+σPE2)(σFTD2+σa2+σPE2+σe2)(σFTD2+σa2+σPE2+σe2).

The same model [1] was used in 3 separate bi-trait models to estimate genetic correlation between lactose and fat, protein, and casein contents. Variance components were estimated using the average information-REML algorithm implemented in the blupf90 family of programs (Misztal et al., 2014). As reported in literature (e.g., Garcia et al., 2020), the combined application of different methodologies to the same objective can enhance detection power. Thus, 2 different approaches were tested to carry out GWAS: (1) back-solving of GEBV and (2) a Bayesian approach. In the first approach, the equivalence between SNP-BLUP and GBLUP (Wang et al., 2012) was exploited by back-solving GEBV into SNP effects; *P*-values for SNP effects were computed according to Aguilar et al. (2019). The significance threshold was computed as 0.05 divided by the number of independent blocks—that is, a Bonferroni correction that accounts for linkage disequilibrium (**LD**) among the SNPs (e.g., Cesarani et al., 2023). The number of independent blocks (n = 1,847) was estimated using PLINK v1.9 (Chang et al., 2015), accounting for LD among markers. This first approach was performed using the BLUPF90 family of programs (Misztal et al., 2014). In the second approach, we used the Bayesian method implemented in BayesR (Moser et al., 2015). A chain with 50,000 samples was adopted, with the first 5,000 being discarded as burn-in. Under this approach, the probability of each marker being assigned to 4 potential effect distributions is estimated: $N\left(0,0\sigma_{a}^{2}\right),$ $N\left(0,0.0001\sigma_{a}^{2}\right),$ $N\left(0,0.001\sigma_{a}^{2}\right),$ and $N\left(0,0.01\sigma_{a}^{2}\right).$ The posterior inclusion probability (**PIP**) of each marker was derived as the sum of the probabilities of inclusion across the 3 nonzero distribution categories. Those SNPs with a PIP greater than 0.30 were deemed significant (Pasam et al., 2017; Manca et al., 2021). Genes mapped within 250 kb downstream and upstream were considered as candidate genes potentially associated with lactose content (Sutera et al., 2021a; Carta et al., 2023).

The average phenotypic values (±SD) were 1.40 ± 0.52 kg for milk yield, 4.47% ± 0.35% for lactose percentage, 7.08% ± 1.06% for fat, 5.82% ± 0.65% for protein, and 4.63% ± 0.54% for casein. The mean lactose percentage was comparable to values reported for other breeds, such as Sarda (Carta et al., 2023), and fell within the range observed across dairy sheep populations (Ferro et al., 2017; Marshall et al., 2025). Using the single-trait model, h^2^ and r^2^ for lactose content were 0.20 ± 0.06 and 0.33 ± 0.03, respectively. The heritability estimates for fat and protein contents obtained in this study are consistent with those reported in the literature for the same breed. For instance, Sutera et al. (2021b) reported heritability values of 0.11 ± 0.03 and 0.15 ± 0.03 for fat and protein percentages, respectively. The contribution of the FTD on the total phenotypic variance was 0.15 ± 0.02. The h^2^ estimate observed in the present study suggests moderate genetic control of lactose content in the Valle del Belice breed. Lower h^2^ (0.10 ± 0.05) and moderate r^2^ (0.42 ± 0.02) were reported in the Sarda sheep breed (Carta et al., 2023). A former study (Selvaggi et al., 2017) applied a sire model to estimate variance components for lactose content in 3 other Italian dairy sheep breeds: heritability estimates were 0.14 ± 0.01 for Comisana, 0.18 ± 0.01 for Leccese, and 0.17 ± 0.02 for Sarda. On the contrary, a recent study in a New Zealand dairy sheep population (Marshall et al., 2025) reported higher estimates for both heritability and repeatability (h^2^ = 0.39; r^2^ = 0.53). In dairy cattle, higher h^2^ values have been reported, ranging from 0.25 to 0.37 (Haile-Mariam and Pryce, 2017). In goats, Bagnicka et al. (2016) reported h^2^ of 0.27 and r^2^ of 0.55 for lactose content. Overall, the estimates obtained in this study are consistent with those reported in other ruminant species and indicate that lactose content is under moderate genetic control.

Although its heritability is generally lower than that of fat and protein percentages, the results suggest that lactose can respond to selection. This is further supported by the genetic CV, which was estimated at 3.46%, indicating the presence of exploitable genetic variability for this trait.

At the same time, the non-negligible contribution of environmental and permanent effects highlights the importance of management conditions in shaping phenotypic expression. As reported in Table 1, the estimated genetic correlations with milk composition traits were not significantly different from zero, likely due to the limited sample size, which reduces the statistical power to accurately estimate genetic correlations and results in relatively large standard errors. Because of that, h^2^ and r^2^ for lactose content remained substantially unchanged in the bivariate analyses, indicating robustness of the model and consistency of variance component partitioning. Similarly, phenotypic correlations between lactose and fat, as well as between lactose and protein were not significantly different from zero. When lactose content was analyzed with milk yield, its heritability increased to 0.26 ± 0.06; between these 2 traits, positive genetic (0.50 ± 0.10) and phenotypic (0.31) correlations were found. Larger genetic correlation between lactose content and milk yield (0.79 ± 0.16) was reported in Sarda dairy sheep (Carta et al., 2023). The phenotypic correlation between lactose and casein content was significant but very low (0.07). Comparable results were reported by Marshall et al. (2025), who found phenotypic and genetic correlations between lactose percentage and other milk traits that were not significantly different from zero (i.e., with 95% CI including zero).

**Table 1:** Results of the bivariate models where lactose content was analyzed with milk yield and fat, protein, and casein contents^1^

Comparison vs. lactose content	Lactose			Second trait			Correlation	
	FTD^2^	h^2^	r^2^	FTD^2^	h^2^	r^2^	Genetic	Phenotypic^2^
Milk yield	0.14 ± 0.02	0.26 ± 0.06	0.35 ± 0.03	0.28 ± 0.03	0.27 ± 0.06	0.35 ± 0.03	0.50 ± 0.11	0.31***
Fat content	0.15 ± 0.02	0.20 ± 0.06	0.33 ± 0.03	0.40 ± 0.04	0.13 ± 0.04	0.22 ± 0.02	−0.15 ± 0.19	0.02^NS^
Protein content	0.15 ± 0.02	0.20 ± 0.06	0.33 ± 0.03	0.38 ± 0.04	0.21 ± 0.05	0.26 ± 0.03	−0.14 ± 0.17	0.02^NS^
Casein content	0.15 ± 0.02	0.20 ± 0.07	0.33 ± 0.03	0.39 ± 0.04	0.21 ± 0.06	0.26 ± 0.03	0.03 ± 0.14	0.07***

1 Values are estimates ± SE.

2 NS = not significant (*P* > 0.05).

*** Highly significant (*P* < 0.001).

To increase the robustness of the results and account for different assumptions on the genetic architecture of the trait, 2 complementary GWAS approaches were applied. The back-solving approach relies on the equivalence between GBLUP and SNP-BLUP and assumes an infinitesimal model with normally distributed SNP effects (Wang et al., 2012), whereas the Bayesian method allows for heterogeneous SNP effect sizes and performs variable selection (Moser et al., 2015). The use of both methods enables the identification of genomic regions consistently detected across different statistical frameworks. Figure 1 shows the Manhattan plot of the GWAS for lactose content in the Valle del Belice breed. When combining the results obtained from the 2 analytical methods (back-solving of GEBV and Bayesian approach), only 1 marker surpassed the predefined significance thresholds (Figure 1), and GWAS results that are characterized by a single outlier marker, in the absence of other associated peaks, are biologically relevant (Kijas et al., 2013; Mastrangelo et al., 2019). This SNP was located on OAR02 at position 33,635,275 bp. The relatively small sample size may have limited the power to detect additional QTLs, particularly those with small effects, and therefore the absence of further significant signals should be interpreted with caution. The proportion of genetic variance explained by the lead SNP was 0.006%, supporting a minor individual effect and a predominantly polygenic architecture.

**Figure 1:**
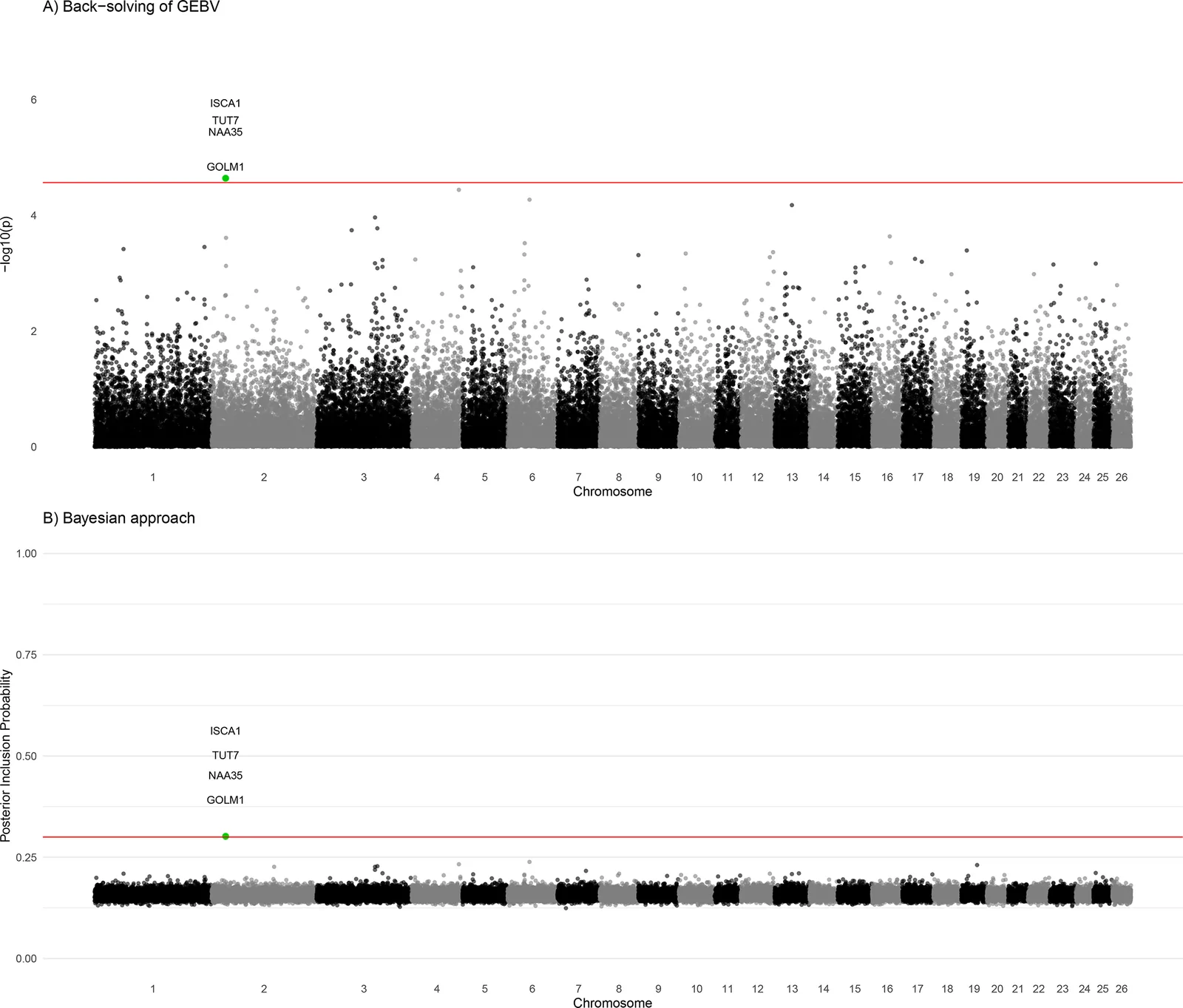
Manhattan plot of the genome-wide association study for lactose content in Valle del Belice sheep using the back-solving of the genomic breeding values (A) or the Bayesian approach (B). The green dot represents the SNP exceeding the significance threshold (red horizontal line) in both approaches.

A search for positional candidate genes within the genomic region surrounding the significant marker led to the identification of 4 annotated genes. The *ISCA1* gene has previously been associated with mitochondrial dysfunctions (Shukla et al., 2017), and it has been reported within selection signature regions in sheep (Mészárosová et al., 2022). The other genes in the region (*TUT7*, *NAA35*, and *GOLM1*) have been linked to immune-related processes and have been identified within runs of homozygosity or selection signatures in cattle (Toro-Ospina et al., 2022; Song et al., 2024). None of these genes are established core components of the lactose synthesis pathway (e.g., the lactose synthase complex). Therefore, the detected association may reflect regulatory mechanisms or broader cellular processes influencing mammary secretory activity rather than direct enzymatic control of lactose biosynthesis. A previous study in sheep (Carta et al., 2023) identified several potential genomic regions associated with variation in lactose percentage. Although these regions did not include canonical genes of the lactose synthesis pathway, several candidate genes were involved in immune response, inflammatory signaling, and mammary gland function. Similarly, GWAS conducted in cattle (Costa et al., 2019b) identified genomic regions harboring genes implicated in intra- and extracellular transport mechanisms, immune response, and mastitis susceptibility. Studies in buffalo (Awad et al., 2020) also reported candidate genes associated with immune function, SCC, and mammary gland health. Collectively, these cross-species findings suggest that genetic variation underlying milk lactose content is not necessarily driven by structural variation in key biosynthetic enzymes but may instead be influenced by genes involved in mammary gland homeostasis, immune response, substrate transport, and secretory efficiency. Although the specific genomic regions differ across species and populations, a recurring theme is the contribution of genes related to mammary gland physiology and immune regulation. Among the positional candidates identified in our study, *GOLM1* (Golgi membrane protein 1) encodes a type II transmembrane protein localized in the *cis* and medial Golgi compartments. It is involved in protein processing, intracellular trafficking, and maintenance of the Golgi structure, contributing to vesicle-mediated transport and cargo sorting (Frappaolo et al., 2020; Spano and Colanzi, 2022). These processes are essential for proper mammary epithelial cell function. Although *GOLM1* is not a structural component of the lactose synthesis machinery (e.g., the lactose synthase complex), its localization within the Golgi apparatus makes it a biologically plausible indirect candidate. Lactose synthesis occurs in the Golgi lumen of mammary epithelial cells and is tightly coupled with vesicle-mediated secretion processes (Shennan and Peaker, 2000; Zhao, 2014). Therefore, proper Golgi organization and intracellular trafficking are essential for enzyme localization, substrate availability, and efficient secretion of milk components. In this context, variation in genes involved in Golgi structure and function may indirectly influence lactose content by affecting secretory efficiency rather than the enzymatic steps of lactose biosynthesis. However, no direct evidence currently links *GOLM1* to the lactose synthase complex or to specific regulation of lactose biosynthesis, and its potential role should therefore be interpreted with caution.

This study provides novel insights into the genetic architecture of lactose content in dairy sheep. The moderate heritability and repeatability estimates indicate that lactose percentage is under meaningful additive genetic control, despite its physiological stability and central role in osmotic regulation. The GWAS identified a single significant genomic region on OAR02, pointing to a locus not directly related to canonical lactose biosynthesis genes but located near genes involved in mitochondrial function, immune response, and intracellular trafficking. These findings align with evidence from other ruminant species, suggesting that genetic variation in lactose content may be mediated by broader regulatory mechanisms influencing mammary gland homeostasis and secretory efficiency rather than by structural variation in the lactose synthase complex. Overall, the results provide a first genomic characterization of lactose content in this breed and highlight its moderate genetic determinism. However, further studies are needed to better understand its relationships with economically important traits, such as udder health and milk coagulation properties, and to evaluate its potential relevance in breeding programs. Validation in larger and independent populations will be essential to confirm the identified genomic region, improve the accuracy of genetic parameter estimates, and assess the generalizability of these findings.
